# Characterization of the Anti-Inflammatory Capacity of IL-10-Producing Neutrophils in Response to *Streptococcus pneumoniae* Infection

**DOI:** 10.3389/fimmu.2021.638917

**Published:** 2021-04-28

**Authors:** Liliana A. González, Felipe Melo-González, Valentina P. Sebastián, Omar P. Vallejos, Loreani P. Noguera, Isidora D. Suazo, Bárbara M. Schultz, Andrés H. Manosalva, Hernán F. Peñaloza, Jorge A. Soto, Dane Parker, Claudia A. Riedel, Pablo A. González, Alexis M. Kalergis, Susan M. Bueno

**Affiliations:** ^1^ Millennium Institute on Immunology and Immunotherapy, Departamento de Genética Molecular y Microbiología, Facultad de Ciencias Biológicas, Pontificia Universidad Católica de Chile, Santiago, Chile; ^2^ Servicio de Anatomía Patológica, Hospital Barros Luco Trudeau, Santiago, Chile; ^3^ Acute Lung Injury Center of Excellence, Division of Pulmonary, Allergy, and Critical Care Medicine, Department of Medicine, University of Pittsburgh, Pittsburgh, PA, United States; ^4^ Department of Pathology, Immunology and Laboratory Medicine, Center for Immunity and Inflammation, Rutgers New Jersey Medical School, Newark, NJ, United States; ^5^ Millennium Institute on Immunology and Immunotherapy, Departamento de Biología Celular, Facultad de Ciencias Biológicas y Facultad de Medicina, Universidad Andrés Bello, Santiago, Chile; ^6^ Departamento de Endocrinología, Facultad de Medicina, Pontificia Universidad Católica de Chile, Santiago, Chile

**Keywords:** pneumonia, *Streptococcus pneumoniae*, interleukin-10, IL-10-producing neutrophils, adoptive neutrophil transfer

## Abstract

Neutrophils are immune cells classically defined as pro-inflammatory effector cells. However, current accumulated evidence indicates that neutrophils have more versatile immune-modulating properties. During acute lung infection with *Streptococcus pneumoniae* in mice, interleukin-10 (IL-10) production is required to temper an excessive lung injury and to improve survival, yet the cellular source of IL-10 and the immunomodulatory role of neutrophils during *S. pneumoniae* infection remain unknown. Here we show that neutrophils are the main myeloid cells that produce IL-10 in the lungs during the first 48 h of infection. Importantly, *in vitro* assays with bone-marrow derived neutrophils confirmed that IL-10 can be induced by these cells by the direct recognition of pneumococcal antigens. *In vivo*, we identified the recruitment of two neutrophil subpopulations in the lungs following infection, which exhibited clear morphological differences and a distinctive profile of IL-10 production at 48 h post-infection. Furthermore, adoptive transfer of neutrophils from WT mice into IL-10 knockout mice (*Il10^-/-^*) fully restored IL-10 production in the lungs and reduced lung histopathology. These results suggest that IL-10 production by neutrophils induced by *S. pneumoniae* limits lung injury and is important to mediate an effective immune response required for host survival.

## Introduction

Pneumonia is defined as an inflammatory condition affecting the lower respiratory tract and is considered the leading cause of death due to infections in children under five years of age (1, 2). *S. pneumoniae*, a Gram-positive encapsulated bacterium, is a major cause of bacterial community-acquired pneumonia and one of the most important causative agents of invasive disease (3, 4). *S. pneumoniae* possesses numerous virulence factors required for a successful colonization of the nasopharynx and for immune evasion (5, 6). Once the immune response and the mucosal barriers of the host become compromised, *S. pneumoniae* disseminates to other organs, causing a wide spectrum of diseases, which range from mild as otitis and sinusitis, to severe such as Invasive Pneumococcal Disease (IPD) (7–9). IPD comprises bacteremic pneumonia, meningitis and sepsis and is considered an important cause of mortality in children and the elderly, representing approximately 12% of deaths every year worldwide (10). Although a consistent decrease in the prevalence of pneumonia in children under five years old has been reported due to immunization programs (11), still 3.7 millions of severe episodes of pneumococcal disease and more than 300,000 deaths in children were estimated worldwide in 2015 (4). Furthermore, lack of effective vaccination programs in developing countries, serotype replacement of those not included in current vaccines and the continuous emergence of antibiotic resistance make *S. pneumoniae* a worldwide concern (12–14).

During *S. pneumoniae* lung infection, the immune response of the host plays a critical role in the evolution of the infection (6, 15). The immune response against *S. pneumoniae* is characterized by the release of several cytokines and chemokines required for the recruitment of neutrophils, monocytes and lymphocytes to the lungs (5), being neutrophils essential for bacterial elimination (16–18). However, given that an appropriate lung function is critical to sustain life in mammals and humans, the immune response against *S. pneumoniae* in the lung must be tightly regulated to guarantee pathogen elimination and, at the same time, prevent excessive inflammation and tissue damage. To guarantee this requirement, anti-inflammatory cytokines, such as interleukin-10, are produced during infection to modulate the lung inflammatory response (19) and restore the host’s immune homeostasis (20, 21). Previous data published by our group showed that IL-10 is strongly induced in the lungs during the first 48 h after pneumococcal exposure (22). Results obtained in this study highlight that the prevention of excessive lung damage by IL-10 is critical to promote host survival, but it also might favor bacterial dissemination (22). Identification of the signals that trigger the early IL-10 production in pneumococcal pneumonia remains unaddressed. However, previous *in vitro* and *ex vivo* assays have shown that *S. pneumoniae* might be involved in the induction of an anti-inflammatory profile in macrophages and dendritic cells, which occurs through cell recognition of CpG motifs in bacterial DNA, pneumolysin, and cell wall components such as peptidoglycan and lipoteichoic acids (23–25). However, until now, it is not clear whether these stimuli and these myeloid cells are directly involved in IL-10 production during the first 48hpi with *S. pneumoniae.* In *Klebsiella pneumoniae*-induced pneumonia, macrophages, monocytic myeloid-derived suppressor cells (M-MDSCs) and neutrophils have been described as the main contributors to IL-10 production during bacterial pneumonia (19, 26, 27). In fact, using a GFP-reporter mouse during *K. pneumoniae* lung infection, neutrophils were identified as IL-10-producing cells in lungs during the first 48 h post-infection (19). These findings are in line with several studies, showing that neutrophils are able to produce IL-10 during infection, indicating that these cells might be playing a role beyond pathogen clearance in pneumonia (28–30).

Conventionally, neutrophils have been considered crucial players in pro-inflammatory processes and short-lived effector cells that react to infections and different inflammatory stimuli (31, 32). That view has changed over the last decade due to the observation that the half-life of neutrophils is on average 5.4 days in humans (33) and by the identification of immunomodulatory and suppressive properties in human and mouse neutrophils, such as IL-10 production (28, 34–36). In fact, there is evidence that anti-inflammatory neutrophils, expanded after exposure to bacterial components and suppressed the pro-inflammatory activity of macrophages, dendritic cells and T cells *in vitro* (30, 37). Based on the potential immunomodulatory role of neutrophils mediated by the production of IL-10, in the present study we evaluated whether the production of IL-10 by neutrophils recruited to the lungs in response to *S. pneumoniae* infection downmodulates inflammation of this tissue and promotes host survival. Using a mouse model of infection, our data shows that neutrophils contribute significantly to IL-10 production during the acute phase of *S. pneumoniae* infection in the lungs, displaying a regulatory role and influencing disease outcome. In addition, given the functional and morphological heterogeneity of neutrophils (38), we characterized the neutrophilic population recruited to the lungs in response to *S. pneumoniae*. Consistently with previously published data from our laboratory, we found two neutrophil subsets present in the lungs. We then characterized them in terms of morphology and their ability to produce IL-10, providing new insights on the functional heterogeneity of neutrophils and their roles during infection.

## Materials and Methods

### Mice

Adult (6-8 weeks) male C57BL/6 (WT), B6.129P2-*Il10^tm1Cgn^*/J (*Il10* ^-/-^), B6(Cg)-*Il10^tm1.1Karp^*/J (IL-10::eGFP) and B6.SJL-*Ptprc^a^ Pepc^b^/*BoyJ (CD45.1) mice were obtained originally from the Jackson Laboratories (Bar Harbor, ME). All mice were co-housed and fed with the same chow for 12 generations in specific pathogen-free conditions in the animal facility (CIBEM) at the Pontificia Universidad Católica de Chile. All experimental protocols were reviewed and approved by the Scientific Ethical Committee for Animal and Environment Care and the Scientific Committee for Research Safety of the Pontificia Universidad Católica de Chile (protocol number 160822002). All animal experiments were performed by trained personnel according to the Guide for Care and Use of Laboratory Animals (39). A clinical score was used to supervise mouse wellness, which includes parameters such as weight loss, pain signs, and respiratory and neurologic changes (**Supplementary Data** and **Table S1**).

### Bacterial Growth and Preparation

*Streptococcus pneumoniae* D39 (NCTC 7466/capsulated) and the non-capsulated derivative R6 (NCTC 13276) strains were grown in Todd Hewitt broth supplemented with 0.5% yeast extract (THYE), without agitation at 37°C/CO_2_ 5%, until an OD_600_ 0.5 was reached. Then, the bacterial suspension was stored in 10% glycerol at -80°C until use. Colony-forming units (CFUs) of bacterial suspensions were determined by serial dilutions on blood agar before the storage and after each assay (22, 40). To check the capsule presence in the D39 strain, we seeded D39 and R6, in order to compare the phenotype of colonies. Before use, bacterial suspensions were centrifuged at 4,700 g for 7 min at 4°C and washed with sterile PBS or 0.5% THYE broth. For infections, the bacterial pellet was resuspended in 0.5% THYE broth. For *in vitro* assays, bacteria were resuspended in sterile PBS and heat-inactivated at 95°C for 15 min.

### Purification of Naïve Neutrophils From Bone-Marrow Cells

Femurs and tibias were obtained from WT and/or *Il10* ^-/-^ mice. Bones were cleaned from muscle and fat residues and bone marrow was flushed by perfusion with RPMI 1640 medium supplemented with 10% FBS and 2mM EDTA. Red blood cells were lysed with a hypotonic solution of NaCl 0.2% and lysis was stopped with an isotonic solution of NaCl 1.6%. Next, cells were counted and stained for negative selection by using a neutrophil purification kit (MACS, Miltenyi Biotech). The purity obtained after neutrophils purification was always ≥96% (**Figure S1**). After purification, neutrophils were diluted in sterile PBS. For *in vitro* assays, neutrophils were placed in HBSS containing Ca^2+^ and Mg^2+^ supplemented with 0.1% gelatin (Sigma- Aldrich G7765).

### Adoptive Transfer Assays and Infections

*Il10* ^-/-^ mice received intranasally 50 μL of sterile PBS containing 1x10^6^ purified neutrophils obtained from WT mice (*Il10* ^-/-^ + WT). Non-transferred *Il10* ^-/-^ mice (*Il10* ^-/-^), non-transferred WT mice (WT) and *Il10* ^-/-^ mice transferred with *Il10* ^-/-^ neutrophils (*Il10* ^-/-^ + *Il10* ^-/-^) were included as control groups. After 24 h, mice were intranasally infected with 3x10^7^ CFUs of *S. pneumoniae* D39. The survival rate, weight loss and clinical score were monitored daily until 10 days post infection (dpi). Mice monitoring was assessed according to **Table S1** and the score was recorded daily before mice processing. Additionally, adoptive transfer assays were also performed to evaluate disease severity parameters such as bacterial loads, lung infiltration by proinflammatory cells and tissue damage, at 48 h post infection (hpi). At least three independent assays were performed.

### Tissue Preparation

Total blood was obtained by cardiac puncture in mice under deep terminal anesthesia. Then, dissection of the trachea was performed in euthanized mice, using sterile surgical material. A cannula was inserted through the trachea and serial washes were performed with 1,500 µl of sterile PBS to obtain bronchoalveolar lavage. Lungs, spleen and brain were extracted with sterile surgical instruments. Lungs were cut into small pieces with sterile scissors and digested in 7 mL of Collagenase IV (1mg/mL) for 1 h at 37°C with agitation. After the incubation period, 7 mL of 5 mM EDTA was added to each tube and mixed gently (this process was exclusively applied for lungs). Then, lungs and the remaining organs were homogenized and passed through a cell strainer (70 μm pore) and recovered in their original tube containing PBS. For bacterial load determination, serial dilutions of each organ were seeded in blood agar plates at 37°C with 5% CO_2_. Bacterial loads were calculated as CFUs per organ. Lungs and BALF pellets were separated by centrifugation during 5 min at 0.4 g. Each pellet was treated with a sterile solution of 1X ammonium chloride-potassium (ACK) for 5 min and then washed with PBS. Cells obtained after this process were stained with antibodies to characterize the inflammatory cell infiltrate by flow cytometry. Cytokine protein levels (IL-6, TNF-α, IFN-γ, IL-10, IL-12p40) were determined in BALF supernatants by ELISA (BD OptEIA).

### Lung Histopathological Analyses

Histopathological analyses were performed in mice of each group at 48 hpi. Lungs were removed and fixed in 4% paraformaldehyde for 48 h, then embedded in paraffin wax blocks and sliced with a microtome in 5 μm sections. Slices were incubated at 60°C for 1 h and stained with Hematoxylin and Eosin (H&E staining). Images from each slide were obtained by using a Leica SCN400F slide scanner and analyzed with the Aperio ImageScope Software. Pathological changes in lungs were blindly evaluated by a pathologist and quantified following the parameters detailed in **Table S2**.

### Flow Cytometry

Analyses of myeloid cells were performed by flow cytometry using the following markers: anti-CD45 (clone 30-F11, BD), anti-CD11b (clone M1/70, BD), anti-CD11c (clone HL3, BD), anti-Ly6C (clone AL-21, BD), anti-Ly6G (clone 1A8, Biolegend), anti-MHC-II (clone M5/114.15.2, BD), anti-Siglec-F (clone E50-2440, BD), anti-CD103 (clone M290, BD), anti-CD64 (clone 10.1, Biolegend), anti-CD45.1 (clone A20, BD), anti-CD31 (clone 390, Biolegend), anti-CD44 (clone IM7, BD), anti-CD62L (clone MEL-14, BD). Dead cells were excluded from analyses using the LIVE/DEAD Fixable Viability Stain 510 (BD). Data were acquired on a Fortessa X-20 flow cytometer (BD Bioscience, Oxford, UK) and analyzed using FACSDiva (BD Bioscience, UK) or FlowJo software (Tree Star, Ashland, Oregon). IL-10 intracellular staining was performed using an anti-IL-10 antibody (clone JES5-16E3, Biolegend). CountBright™ absolute counting beads (Invitrogen™ C36950) were added to samples before analyses and used to calculate the total number of cell populations per μL. Each organ was diluted in 1mL before the staining and 200 μL from each sample was mixed with 50 μL of beads solution. Quantification was performed according to manufacturer’s indications. Cell type identification was performed according to the gating strategy showed in the supplementary material (**Figure S2**). IL-10 expression data were calculated according to eGFP expression represented by fluorescence in the FITC channel. Additionally, the basal eGFP expression by each cell population was determined using an uninfected WT (eGFP^neg^) mice (**Figure S3**).

### Neutrophil Characterization by Flow Cytometry and Transmission Electron Microscopy

IL-10::eGFP mice were intranasally instilled with vehicle (uninfected) or 3x10^7^ CFUs of *S. pneumoniae*. Lungs and bronchoalveolar lavage (BALF) were obtained from both groups after 12, 24 and 48 hpi. Lungs were processed and stained for flow cytometry. Cells obtained from 24 hpi BALFs were stained and sorted by using a BD FACS Aria II (BD Biosciences, San Jose, USA). CD11b^+^CD11c^-^Ly6G^+^ cells were selected and subsequently separated by size (Forward scatter). FACS was conducted at the Flow Cytometry Facility of the Pontificia Universidad Católica de Chile. Sorted neutrophil subsets were fixed by immersion in 2.5% glutaraldehyde and subsequently treated and prepared at the Advanced Microscopy Facility of the Pontificia Universidad Católica de Chile. Sections of 80 nm of diameter were obtained using an ultramicrotome Leica Ultracut R. Grids were visualized and photographed at 8200X in a Phillips Tecnai 12 electron microscope at 80 kV. TEM microphotographs were analyzed with Image-J software. Morphological features evaluated for each neutrophil subtype included size, euchromatin fraction, quantification of cytoplasmic structures and cytoplasm electrodensity. Each cell characteristic was evaluated according to previously reported parameters (41). This assay was repeated twice.

### *In Vitro* Assays for the Evaluation of IL-10 Production by Neutrophils

Bone marrow-derived neutrophils were incubated with different stimuli during 1 h in constant agitation at 37°C in 5% CO_2_ and subsequently incubated for 23 h. Cells were recovered for counting and viability evaluation by flow cytometry. Supernatants were stored at -80°C and then evaluated for IL-10 detection by ELISA. Bacteria used in these assays were inactivated by heat at 90°C for 15 min. Heat-killed bacteria were seeded in blood agar to confirm bacterial inactivation. The assays were performed with three different multiplicity of infection (MOIs) of heat-killed *S. pneumoniae* (MOI 25, 50, 100) to neutrophils. The experiments were also performed with BALFs obtained from mice intranasally infected or instilled with vehicle. BALF was obtained 24 h after the instillation. Subsequently, BALF was centrifugated and separated, and supernatants were stored at -80°C until use. Additionally, neutrophils were exposed to DNA obtained from *S. pneumoniae* D39 (MOI 25 and MOI 50). Some experiments were performed with pneumococcal purified cell wall obtained according to the protocol used by Fan and Jin^1^. At least three interdependent assays were performed.

### Statistical Analyses

All the experiments contain data from two or three independent experiments and each assay was performed with at least 2 animals per group. D’agostino & Pearson Omnibus and Shapiro-Wilk normality tests were performed to confirm the normal distribution of data. Kruskal-Wallis tests with Dunn’s multiple comparison post-tests were performed to analyze the IL-10-producing cells at 24 and 48 hpi, to compare the phenotypical characteristics of FSC^low^ and FSC^high^ subsets evaluated by TEM and data obtained from the IL-10 expression and production in the *in vitro* assays. One-way ANOVA with Sidak’s multiple comparison post-tests were also performed in adoptive transfer assays, in order to compare all the experimental groups with each other in analyses related to lung infiltration, bacterial loads in organs and cytokine determination in BALFs. Kruskal-Wallis with Dunn’s post-test were performed when the sample size was too small or with non-parametric data. Survival curves were compared using a log rank test. Two-way ANOVA and Sidak’s multiple comparisons post-test were performed to analyze the clinical score and the expression of IL-10 and surface markers by neutrophils. In all cases, a P value of <0.05 were considered statistically significant. Statistical analyses were performed using GraphPad Prism 5 for Mac, version 5.01. All data were expressed as means plus standard error (SEM).

## Results

### Neutrophils Contribute to IL-10 Production at Early Times During Pneumococcal Pneumonia

**Figure 1:**
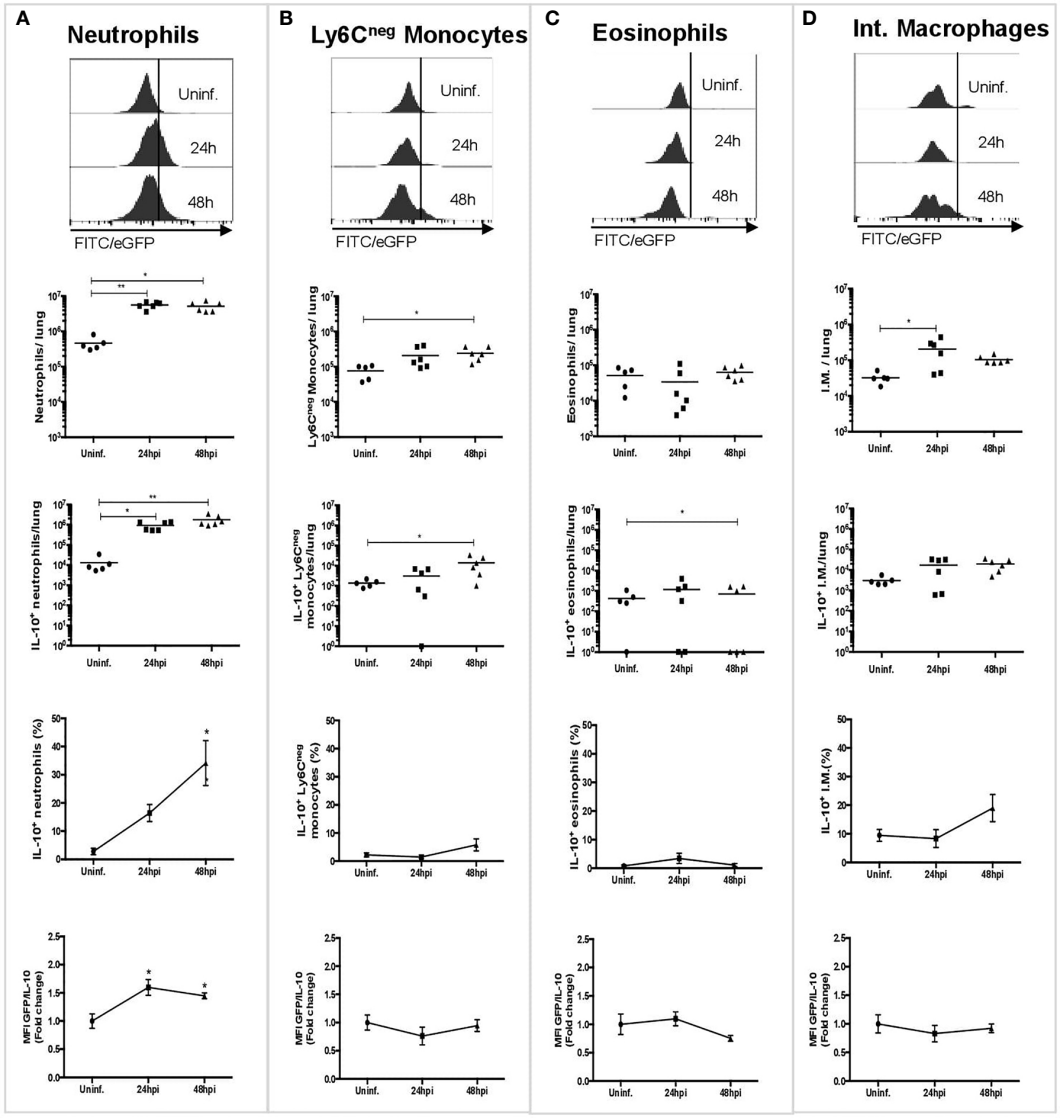
Neutrophils are the main source of IL-10 during the early phase of *S. pneumoniae* infection. Representative histograms and graphs showing the number of cells expressing IL-10 in lungs in uninfected mice (n = 5), and mice infected after 24 (n = 6) and 48 hpi (n = 6), and their corresponding MFI measurements. **(A)** Neutrophils. **(B)** Ly6C^neg^ monocytes. **(C)** Eosinophils. **(D)** Interstitial macrophages. WT uninfected mice were used to establish the baseline of GFP expression (**Figure S3**). Quantification of each cell population was performed using counting beads. Statistics: Kruskal-Wallis with Dunn’s multiple comparison post-test. *P < 0.05; **P < 0.01. Comparisons of the mean of infected and uninfected mice, 24 and 48 hpi. hpi, h post-infection; MFI, Median Fluorescence Intensity; I.M., Interstitial macrophages.

To assess which cells are involved in IL-10 production in the lungs during the acute phase of pneumococcal pneumonia, three experimental groups of IL-10::eGFP mice were either instilled with THYE or infected with *S. pneumoniae* for 24 h or 48 h. Lung cells were stained for flow cytometry characterization with an antibody panel designed to identify dendritic cells (DCs), alveolar macrophages, monocytes, interstitial macrophages, eosinophils and neutrophils (**Figure S2**). Furthermore, quantification of IL-10-production by each cell population was performed by analyzing the expression of eGFP (**Figure 1**). Our findings indicate that neutrophils are the most abundant cells that produced IL-10 either at 24 or 48 h post-infection (**Figure 1A**). Ly6C^neg^ monocytes and eosinophils showed a moderate IL-10 production at 48 h (**Figures 1B, C**). Interstitial macrophages also showed a non-significant upward trend of IL-10^+^ production (**Figure 1D**). Finally, resident alveolar macrophages, interstitial macrophages, monocyte-derived macrophages, Ly6C^+^ monocytes and DCs did not contribute to IL-10 production during *S. pneumoniae* infection (**Figure S4**). IL-10 expression in neutrophils following *S. pneumoniae* infection was further confirmed by intracellular staining with an anti-IL-10 antibody (**Figure S5**). Overall, our data show that neutrophils are the main source of IL-10 among myeloid cells in the lungs during *S. pneumoniae* infection.

### Neutrophils Detected in the Lungs During *S. pneumoniae* Infection Differ in Morphology and IL-10 Production

**Figure 2:**
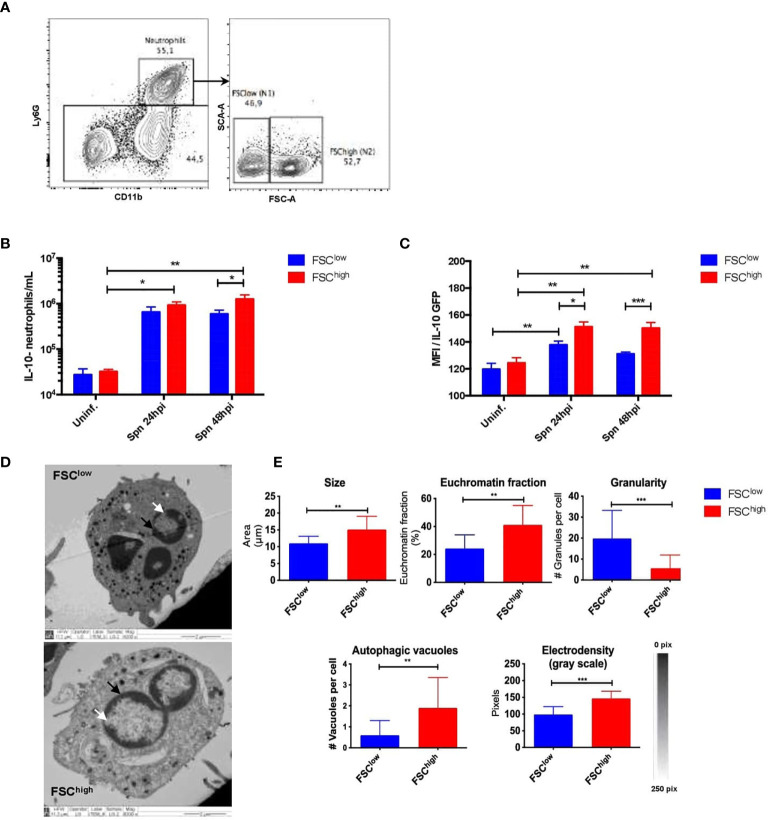
Neutrophil subsets isolated from infected mice exhibit structural and functional differences. **(A)** Representative flow cytometry plots of lung cells show neutrophils gated as CD11b^+^Ly6G^+^ cells. Neutrophil subsets were gated according to FSC parameters as FSC^low^ and FSC^high^. **(B)** Number of FSC^low^ (blue bar) and FSC^high^ (red bar) neutrophils expressing IL-10 in uninfected mice together with the observed neutrophils at 24 and 48hpi. **(C)** IL-10 expression of neutrophils (from IL-10::eGFP mice subsets in lungs at 24 and 48hpi. **(D)** Representative TEM images of FSC^low^ and FSC^high^ subsets obtained from 24h infected lungs. The white arrows point to the heterochromatin and the black arrows point to the euchromatin fraction. **(E)** Phenotypic parameters measured in FSC^low^ and FSC^high^ populations with ImageJ software. Statistics: Tukey’s test following two-way ANOVA and a two-tailed T-test. *P < 0.05; **P < 0.01; ***P < 0.001. TEM, Transmission Electron Microscopy; hpi, hours post-infection; Uninf, Uninfected.

In a previous study, we described the presence of two neutrophil subsets in the lungs with marked differences in size (38). Both of these subsets were rapidly recruited to the lungs during pneumococcal infection, although their biological relevance or their morphological properties were not studied (38). Here, we confirmed the presence of these two subsets by flow cytometry in *S. pneumoniae*-infected and uninfected mice. We named these subsets as FSC^low^ (N1: smaller size) and FSC^high^ (N2: larger size) (**Figure 2A**). Then, we aimed to determine whether these populations play different roles during pneumococcal infection. We first evaluated the expression of maturity and activation surface markers during the first 48 hpi, but no significant differences in the expression of surface markers in both populations were observed (**Figure S6**). Next, we evaluated the ability of each of these subsets to produce IL-10. We observed that FSC^high^ neutrophils significantly produced higher levels of IL-10 at 24 and 48 hpi as compared to FSC^low^ neutrophils, which only showed a mild increase of IL-10 production at 24 hpi (**Figures 2B, C**). An additional morphological characterization of identified neutrophil subsets was performed by TEM in neutrophils sorted by size, using fluorescence activated cell sorting (FACS). Our results show that purified neutrophil populations have phenotypic differences in the shape of the nucleus and chromatin condensation, number of granules and autophagy-related structures (**Figure 2D**). Specifically, FSC^high^ neutrophils exhibited a nucleus with decondensed chromatin, while the FSC^low^ subset had a nucleus predominantly constituted by heterochromatin. Moreover, we also observed differences related to the number of intracellular granules present in the cytoplasm as well as the presence of autophagy-related structures, particularly in the FSC^high^ population **(** **Figure 2E** **).** Together, these data show that the neutrophil population present in lungs in response to any stimulus is a heterogeneous population composed by two subsets that have major morphological differences and are characterized by a differential ability to produce IL-10 *in vivo* in response to *S. pneumoniae*.

### *S. pneumoniae* Directly Triggers IL-10 Production by Neutrophils *in Vitro*

**Figure 3:**
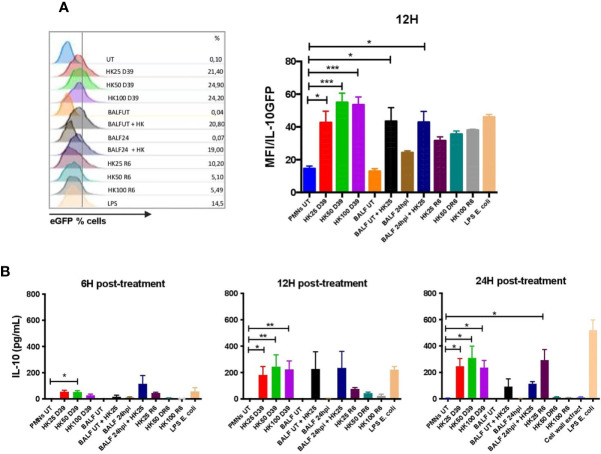
*S. pneumoniae* induces IL-10 production by neutrophils *in vitro*. Bone marrow-derived neutrophils were incubated with different MOIs of heat-killed *S. pneumoniae* (D39 and R6 strains) to determine IL-10 production in response to stimulus. **(A)** Histograms showing the percentage of IL-10 neutrophils by treatment, as well as the eGFP/MFI expression evaluated by flow cytometry at 12hpi. **(B)** Supernatants were recovered and evaluated for IL-10 detection by ELISA at 6, 12 and 24hpi. Kruskal-Wallis with Dunn’s multiple comparison post-tests were performed for statistical analyses. *P < 0.05; **P < 0.01; ***P < 0.001 in each treatment comparison with the untreated cells. Three independent experiments.

In order to elucidate the nature of the stimuli that triggers IL-10 production in neutrophils, we performed *in vitro* assays with bone-marrow neutrophils obtained from IL-10::eGFP mice. These neutrophils were incubated with heat-killed bacteria (MOI 25, 50 and 100) and BALFs obtained from infected and non-infected WT mice. We included in these experiments two *S. pneumoniae* strains: capsulated and non-capsulated. Additionally, BALFs mixed with heat-killed bacteria were included as a treatment. After 12 h, eGFP fluorescence was measured in cells by flow cytometry and IL-10 production was measured by ELISA in the supernatant from cultured neutrophils after 6, 12 and 24 h post-treatment. Results showed that only heat-killed *S. pneumoniae*, but not a soluble factor present in the BALFs from either infected or uninfected mice, stimulated eGFP expression in neutrophils at 12 h post-treatment (**Figure 3A**). These results were confirmed by ELISA, where IL-10 in the supernatant was detected only in neutrophils stimulated with capsulated bacterium (**Figure 3B**). Additionally, we observed IL-10 production 24h post-treatment in neutrophils infected with the non-capsulated strain (MOI 25). However, at 24 h, no IL-10 production was observed in neutrophils infected with higher MOI of the non-capsulated strain (**Figure 3B**). In addition, we stimulated naïve neutrophils with DNA (data not shown) and cell wall extract (peptidoglycan) purified from *S. pneumoniae* D39. Our data show that cell wall extract did not induced IL-10 production *in vitro* (**Figure 3B**). These *in vitro* data suggest that IL-10 expression in neutrophils is directly induced by *S. pneumoniae* PAMPs present in the bacterial surface but not by soluble host or bacterial factors secreted to the airways during infection.

### Adoptive Neutrophil Transfer Improves Survival and Clinical Signs of *Il10* ^-/-^ Mice, Independently of IL-10 Production by Transferred Cells

**Figure 4:**
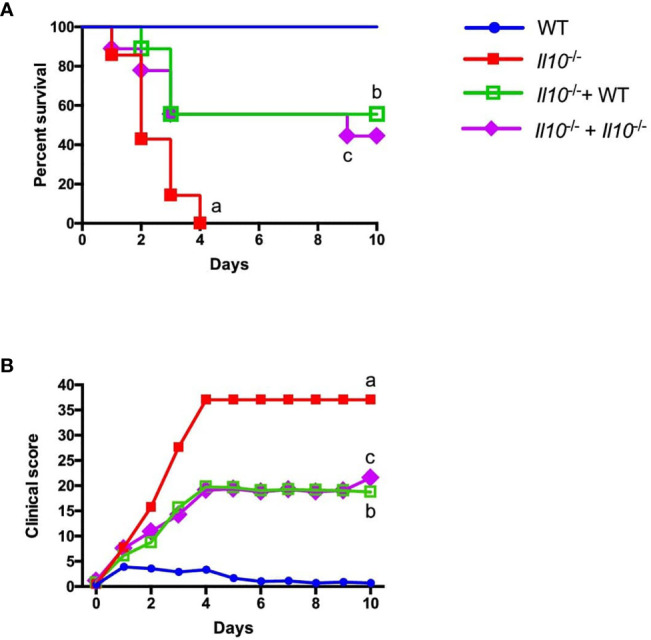
Adoptive transfer of neutrophil improves *Il10* ^-/-^ mice survival and clinical score independently of IL-10 production. Neutrophils obtained from WT and *Il10* ^-/-^ mice were intranasally transferred to *Il10* ^-/-^ mice and 24 h later infected with 3x10^7^ cfu of *S. pneumoniae* (n = 7 to 9 for each group). This experimental design was performed to evaluate the survival and clinical parameters of mice in the different groups during 10 days. **(A)** Survival curve **(B)** Disease parameters were evaluated according to a clinical score guideline including activity, weight loss, body posture and general appearance (**Table S1**). Statistics: Log-rank (Mantel-Cox) test. (a) ****P < 0.0001 between WT and *Il10* ^-/-^, (b) **P < 0.01 between *Il10* ^/-^ and *Il10* ^-/-^+ WT, (c) *P < 0.05 between *Il10* ^-/-^ and *Il10* ^-/-^+ *Il10* ^-/-^. Two-way ANOVA with Tukey’s multiple comparisons post-test were performed. (a) ****P < 0.0001 between WT and Il10^-/-^, (b) **P < 0.01 between Il10^/-^ and Il10^-/-+^ WT, (c) *P < 0.05 between Il10^-/-^ and Il10^-/-+^ Il10^-/-^.

A previous study from our laboratory demonstrated that IL-10 plays a key role in mice survival during *S. pneumoniae* (22). Considering this, we aimed to perform neutrophil adoptive transfer assays into *Il10* ^-/-^ mice (highly susceptible to *S. pneumoniae* infection) to evaluate the impact of the IL-10 produced by neutrophils in mice survival. We transferred bone marrow-isolated neutrophils from WT or *Il10* ^-/-^ donor mice to *Il10* ^-/-^ recipient mice one day prior *S. pneumoniae* infection (day -1). Survival rate and clinical parameters were evaluated over the course of ten days after infection. Our data showed that whereas all non-transferred WT mice survived until the end of the experiment and had a low clinical score, non-transferred *Il10* ^-/-^ mice had the highest clinical score and 100% mortality at day 4 post-infection. Surprisingly, recipient *Il10* ^-/-^ mice transferred with either WT or *Il10^-/-^* neutrophils improved their survival rate up to 60% (**Figure 4**). The increased survival of individuals at the endpoint of the curve was similar in both groups of *Il10^-/-^* transferred mice and no significant differences were observed at any point in survival or score curves between these two groups. To further evaluate whether early transfer of neutrophils to *Il10^-/-^* mice improve their defense against *S. pneumoniae*, bone marrow neutrophils were isolated from IL-10::eGFP mice and subsequently stimulated for 12 h with heat-killed *S. pneumoniae* (MOI=25), to induce IL-10 production. Prior to adoptive transfer, we assessed IL-10 expression and viability by flow cytometry. At the time of transfer, 10-20% of neutrophils were IL-10^+^ with a viability of 80% (**Figure S7A**). The transfer of these cells at 12 hpi resulted in an outcome similar to the one observed in mice transferred with neutrophils previous infection (**Figures S7B, S7C**). Moreover, at the end of the experiment (day 10), we observed that *Il10* ^-/-^ mice that received *Il10* ^-/-^ neutrophils had higher numbers of neutrophils in the lungs as compared to *Il10^-/-^* mice that received IL-10::eGFP neutrophils (**Figure S7D**). These findings support the notion that early activation of neutrophils observed in recipient *Il10^-/-^* mice positively affect disease outcome and host survival, in a mechanism that is independent on IL-10 production.

### Adoptive Transfer of IL-10-Producing Neutrophils to *Il10^-/-^* Recipient Mice Reduced Lung Histopathology, but Impaired Bacterial Clearance of *S. pneumoniae*

**Figure 5:**
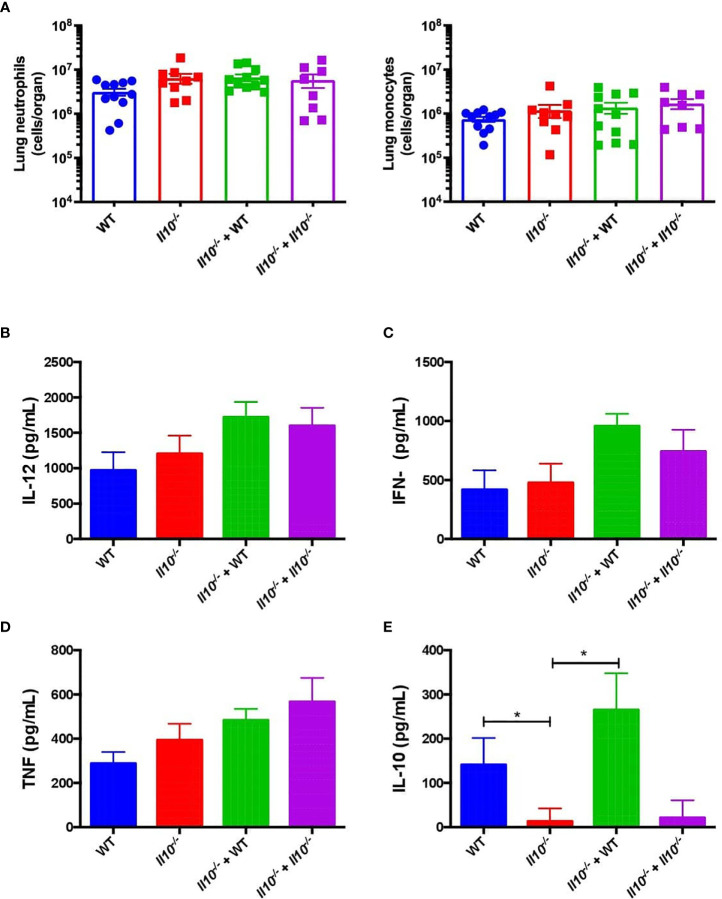
IL-10 produced by neutrophils does not have impact in reduction of pro-inflammatory cytokines or cells infiltration in *Il10* ^-/-^ mice. **(A)** Viable monocyte and neutrophil infiltration in mouse lungs of each group were measured at 48hpi by flow cytometry. Pro-inflammatory **(B–D)** and anti-inflammatory **(E)** cytokines obtained from BALFs at 48hpi were determined by ELISA in each control and transferred group. Statistics: One-way ANOVA with Sidak’s multiple comparison post-test. *P < 0.05. Comparisons of the mean of each group with the mean of every other groups. n = 9 to 12 per group. BALFs, Bronchoalveolar Lavages; hpi, hours post infection.

We next evaluated the effect of neutrophil-produced IL-10 in monocyte and neutrophil recruitment, cytokine production, bacterial burden and histopathological changes in recipient mice at 48 hpi. The analyses of lung infiltration by flow cytometry showed no significant differences in the number of live neutrophils and monocytes in lungs at 48 hpi among the experimental groups (**Figure 5A**). However, our data shows that transfer of WT neutrophils fully restored the production of IL-10 in the BALF of *Il10* ^-/-^ recipient mice at 48 h, although it did not have a major effect in the production of important pro-inflammatory cytokines, such as IL-12, IFN-γ and TNF-α in the BALFs (**Figures 5B–E**). To determine that transferred neutrophils remained in the respiratory tract, 1 x 10^6^ bone marrow-derived neutrophils from CD45.1 mouse were transferred into CD45.2 congenic recipient mice. Recipient mice were infected 1-day post transfer and at 24 hpi lungs, BALF, blood and spleen were harvested to determine neutrophil percentages from CD45.1 and CD45.2. As expected, transferred neutrophils in the BALF represented 10% of total neutrophils. In the lung tissue, this percentage was around 2-4% **(** **Figure S8**). We also evaluated the expression of some homing markers previously evaluated in lung infiltration (CD31, CD44 and CD62L) (42–44) **(** **Figure S9**). CD31 was significantly expressed in CD45.1 neutrophils obtained from lungs, particularly from infected mice, which is consistent with data previously reported for neutrophils in lung undergoing inflammation (44). CD44 is augmented in BALF, indicating that bone marrow-derived neutrophils change the expression of surface markers in response to infection, even when they are intranasally delivered and come from the bone marrow. Finally, the CD62L marker showed low expression in lungs and BALFs, while it was highly expressed in blood **(** **Figure S9** **),** which is in agreement with previous reports (42). These results show that intranasally transferred bone marrow-derived neutrophils remained in the lungs for at least 48 h after transfer. Further, homing data suggest that an important quantity of transferred neutrophils remained in the respiratory tract.

**Figure 6:**
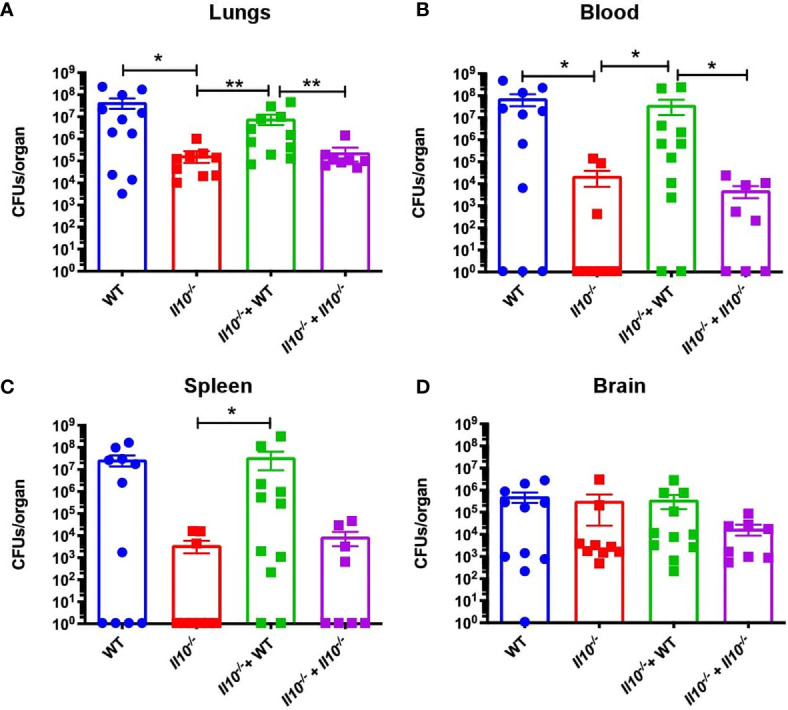
Transfer of WT neutrophils impairs bacterial clearance. Bacterial loads were measured at 48hpi in **(A)** lungs, **(B)** blood, **(C)** spleen and **(D)** brain. Data is shown as CFU per organ. Statistics: T-test with Mann-Whitney post-test. *P < 0.05; **P < 0.01. Comparisons of the mean of each group with the mean of all the other groups. n = 9 to 12 for each group. hpi, hours post-infection; CFUs, Colony Forming Units.

**Figure 7:**
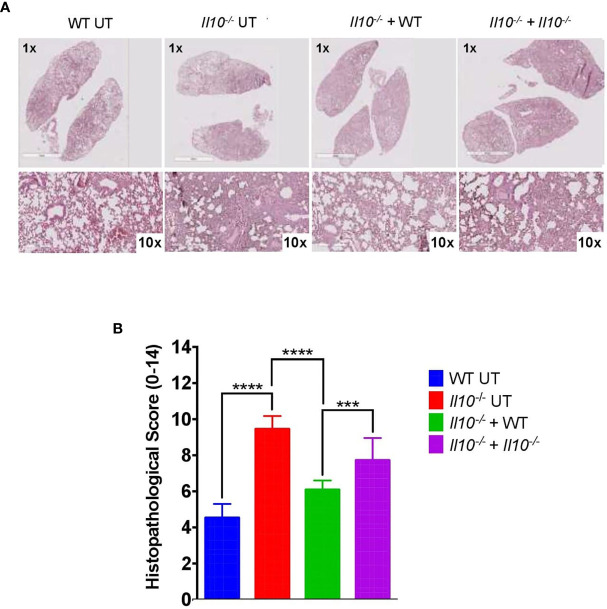
Adoptive transfer of neutrophils from WT to *Il10* ^-/-^ mice induces a decrease in the histopathological score at 48 hpi. **(A)** Representative lung tissue sections stained with H&E at 1X and 10X magnification. These images were used to evaluate histopathological changes after neutrophils adoptive transfer. **(B)** Histopathological score for lung damage. This score was used to quantify lung damage after neutrophils adoptive transfer. To capture photographs and analyze them, the Aperio ImageScope Software and GraphPad Prism Software were used. Statistics: Two-way ANOVA with Tukey’s multiple comparisons post-test were performed. ***P < 0.001; ****P < 0.0001.

We also observed that, as compared to non-transferred *Il10* ^-/-^ or *Il10* ^-/-^ mice transferred with *Il10* ^-/-^ neutrophils, *Il10* ^-/-^ transferred with WT neutrophils showed significantly higher bacterial burden, which was equivalent to WT mice (**Figure 6A**). Moreover, a higher percentage of *Il10* ^-/-^ transferred mice with WT neutrophils presented bacterial dissemination, expressed as significantly higher bacterial burden in blood and spleen (**Figures 6B, C**). No differences among bacterial loads in mice brains were observed in any of the groups (**Figure 6D**). Importantly, our data show that transfer of WT neutrophils to *Il10* ^-/-^ mice reduced lung damage after 48 hpi and significantly protected the alveolar integrity from structural disruptions and hemorrhage, as compared to non-transferred *Il10* ^-/-^ mice (**Figure 7A**). Histopathological observations were scored according to the level of pro-inflammatory cell infiltration, hemorrhage incidence, loss of lung architecture and alveolar structure, and the extension of damage in both lungs (**Supplementary data**, **Table S2**). Results obtained from this analysis showed that *Il10* ^-/-^ mice transferred with WT neutrophils had significantly lesser total score as compared to the other *Il10* ^-/-^ groups (**Figure 7B**). Statistical analysis applied individually per parameter did not show significant differences among groups. However, it is possible to observe that the severity of alveolar wall swelling and lung damage in *Il10^-/^* ^-^+ WT mice were similar to the observed in WT mice (**Figure S10**).

## Discussion

Neutrophils have been recently described as important actors of both innate and adaptive immunity due to their plasticity and immunomodulatory properties, including the ability to produce IL-10 during several inflammatory conditions (28, 45, 46). The modulatory role of neutrophils has been described during sepsis (47, 48) and in chronic infectious diseases, such as tuberculosis and Chagas (29, 49). In agreement with the literature, here we report the existence of heterogeneous population of neutrophils that produce IL-10 in the lungs during *S. pneumoniae* infection. We observed that among the myeloid cells recruited to the lungs, only neutrophils showed a significant production of IL-10 (**Figure 1A**). The high number of IL-10-producing neutrophils found at 24 and 48 hpi suggests that these cells are key components in the prevention of exacerbated inflammatory response during the early phase of *S. pneumoniae* lung infection. It is important to note that the involvement of neutrophils in IL-10 production is different among the broad range of pathogens able to cause pneumonia. For example, in viral pneumonia and tuberculosis, neutrophils are suppliers of IL-10 but the substantial production of this cytokine is produced by interstitial macrophages and T cells (50–52). In the case of lung infection caused by *Klebsiella pneumoniae*, M-MDSCs have the leading role in regulation mediated by IL-10 production (19). This background contrasts with our findings and lead us to propose that the role of neutrophils as the dominant source of IL-10 production is a distinctive feature of pneumococcal pneumonia.

Since *S. pneumoniae* induces a high influx of neutrophils into the lungs that subsequently would produce IL-10, this could be a host mechanism to prevent inflammatory damage activated by bacterial infection (21, 23, 53). Considering this possibility, a major aim of this study was to elucidate whether the production of IL-10 by neutrophils is triggered directly by *S. pneumoniae* or whether is mediated by host factors released in response to the infection. It has been reported that components of the cell wall of *S. pneumoniae* such as peptidoglycan, lipoteichoic acid and lipoproteins induce IL-10 production in macrophages and monocytes after their recognition by Toll-like receptors 2 (TLR2) and 4 (TLR4) (23, 53, 54). TLR2 and TLR4 are widely expressed in the surface of neutrophils and are important mediators of IL-10 production by these cells after ligand binding (28, 55, 56). Here, we provide evidence that neutrophils directly produce IL-10 after *S. pneumoniae* recognition. First, we observed that neutrophils significantly expressed IL-10 during *S. pneumoniae* lung infection *in vivo*, and then we confirmed these findings with *in vitro* assays. Our data show that IL-10 levels in the supernatant were significantly higher when neutrophils were stimulated with encapsulated *S. pneumoniae* as compared to neutrophils stimulated with the non-capsulated *S. pneumoniae*. The different abilities observed for these strains to induce IL-10 production by neutrophils are possible related to the capsule expression. Probably, non-capsulated strain expresses surface PAMPs that promote a pro-inflammatory profile in neutrophils, and higher bacterial loads could increase the exposure to these PAMPs. This effect could be favoring the establishment of an inflammatory response in neutrophils, blocking the pathways related to the production of IL-10. It is also possible that the capsule could be playing a role in coating the pro-inflammatory PAMPs, allowing only the exposition of molecules that might be stimulating IL-10 pathways in neutrophils due to the polysaccharides present in the pneumococcal capsule, which might be activating the paths necessary to activate an anti-inflammatory response. This point has not been evaluated in *S. pneumoniae*, but recently it has been reported for polysaccharide A present in the capsule of *Bacteroides fragilis* (57, 58). Until now, all of these hypotheses have not been addressed experimentally and further studies are required to clarify which bacterial components are the most relevant at inducing IL-10 production. However, based on our results, we suggest that molecular components of the capsule, such as lipoproteins and even capsule polysaccharides, might be potential inducers of IL-10 in neutrophils.

Recently, the discovery of novel roles for neutrophils has been accompanied by phenotypic and functional heterogeneity, which has led to the appearance of new subtypes of neutrophils (59). These subsets have been described mainly in cancer and some infectious diseases (37). However, data regarding neutrophil characterization during *Streptococcus pneumoniae* infection is still recent. After the evaluation of the contribution of neutrophils to IL-10-production during pneumococcal pneumonia, we characterized two neutrophil subsets based on their difference in size (FSC^low^: small or N1; FSC^high^: large or N2). Further, flow cytometry analyses performed to evaluate differences in IL-10 production showed a significant difference in IL-10 expression between FSC^low^ and FSC^high^ at 48 hpi only in infected mice. This result is in line with a recent study, where different neutrophils subsets were described in blood and BALFs obtained from children with viral pneumonia (36).

The characterization of neutrophil subtypes has been established based on differences in surface markers, or cytokine and chemokine profile (60). However, neutrophil plasticity is also supported by other phenotypical characteristics (41, 46). In this context, we performed analyses based on images obtained by transmission electron microscopy. We observed that FSC^low^ cells are small, with a considerably high number of cytoplasmic granules, a low number of vacuoles and a nucleus mainly constituted by heterochromatin. On the other hand, FSC^high^ neutrophils are larger cells, have low presence -or absence- of granules and have presence of multiple vacuoles. Furthermore, FSC^high^ neutrophils present a significant euchromatin fraction, suggesting important changes in gene expression which might explain their differences in IL-10 production, as previously reported in macrophages (61). According to these morphological findings, FSC^low^ and FSC^high^ subtypes display similar characteristics to the ones observed in neutrophil subtypes reported in a systemic inflammatory syndrome in response to intravenous *Staphylococcus aureus* infection (62). Tsuda and colleagues reported the presence of two neutrophil subtypes, the first defined as PMN-I which appeared as small cells with marked pro-inflammatory and cytotoxic properties, and the second named PMN-II, which looked larger and displayed anti-inflammatory properties, characterized by the expression of IL-10 (62). Similarly, FSC^low^ and FSC^high^ subtypes have also been described in cancer, and have been associated with anti- and pro-tumor properties, respectively (34).

Noteworthy, in our study we describe that both neutrophil subsets have the ability to produce IL-10. In this line, our results could be controversial according to the research performed in cancer and systemic infection models, where FSC^low^ like-cells show a very limited ability to secrete IL-10 (37). In this sense it is important to emphasize that experimental conditions used in our study are completely different to those present in a tumor environment or blood, where probably the neutrophil phenotype is conditioned by their anatomical location (46, 63, 64). Additionally, most studies characterizing neutrophil phenotypes are based in the results obtained from *in vitro* tests, which do not allow to evaluate the effect of the “cell-cell” and microenvironment interaction occurring *in vivo*, causing biases to the final phenotype observed (34, 65). On the contrary, here we provide new insights of neutrophil characteristics in the early stage of pneumococcal pneumonia, where IL-10-producing neutrophils have not been described previously. Moreover, according to the results obtained about the ability of both subsets to produce IL-10, we could speculate that FSC^high^ cells are not a different type of cell, but represent a late stage of FSC^low^ cells, which already have the ability to produce IL-10 (66). This fact has been previously discussed, where other authors have reported that “bigger” neutrophils might correspond to cells experiencing an aging process or even might be cells going into apoptosis (30, 67). However, further complementary analyses based on single-cell sequencing would be required to confirm that FSClow and FSChigh are different cell subsets and to find whether they are playing differential roles during pneumococcal lung infection.

This study also comprised adoptive transfer assays to determine the impact of neutrophils able to produce IL-10 in a murine model of pneumonia. Neutrophil adoptive assays have previously been described in other inflammatory processes (45, 68), however our work is the first implementing this assay in pneumococcal pneumonia, based on a protocol previously carried out in our laboratory (19). Here we observed that transferred WT neutrophils restored IL-10 levels in BALF to similar levels observed in control WT mice, reinforcing the idea that neutrophils are an important source of IL-10 during pneumococcal pneumonia. Apparently, this IL-10 levels did not show a clear impact in lung infiltration with pro-inflammatory cells as observed in cytometry analyses. Regarding this point, the number of neutrophils in the lungs of untransferred *Il10* ^-/-^ mice is around 1x10^7^ neutrophils per lung. Considering that the assay performed required 1x10^6^ neutrophils per mouse, which represent around of 10% of the total neutrophils found in lungs during pneumococcal infection, it is possible that the amount of IL-10 producing neutrophils required to prevent inflammatory infiltration is higher. We confirmed this hypothesis by adoptively transferring 1x10^6^ bone marrow-derived-neutrophils from CD45.1 mouse into CD45.2 congenic recipient mice. This assay confirmed that the proportion of donor neutrophils that remained in lungs corresponds to less than a 10% of the total neutrophils observed in lungs during pneumococcal infection and 48 h post-transfer.

Importantly, the IL-10 provided by WT neutrophils to *Il10* ^-/-^ mice had a negative effect in their ability to control bacterial growth and dissemination. Additionally, our results also indicate that IL-10 from WT neutrophils had a great impact in preventing alterations in lung tissue. Histopathological changes were evident in *Il10* ^-/-^ mice after WT neutrophil transfer, which exhibited less damage and better lung architecture as compared to the other *Il10* ^-/-^ experimental groups. These results indicate that the regulatory capacities of neutrophils related to IL-10 production seem to be determinant in the course of pneumococcal murine infection both at a local and systemic level, reinforcing the described dual role of IL-10 in infections (69). Next, to determine the impact of IL-10 provided by neutrophils in mouse survival, we performed adoptive transfer assays followed by a 10-days survival curve. In this experiment we obtained an increased survival in *Il10* ^-/-^ mice that received neutrophils, independent of their capacity to produce IL-10 (**Figure 4**). Our data suggest that the increased survival in *Il10* ^-/-^ mice transferred with WT or *Il10* ^-/-^ neutrophils is due by the sole addition of naïve bone marrow-derived neutrophils directly to the lungs. To evaluate whether the early transfer of neutrophils to *Il10^-/-^* mice improved their defense against *S. pneumoniae* independently on the production of IL-10, we changed the protocol and this time we transferred pre-stimulated IL-10-producing neutrophils 12 hpi. Taken together, these results suggest that the solely presence of naïve transferred neutrophils modulates the host response due to alternative mechanisms not evaluated in this study. These mechanisms may involve an improved microbicidal effect by transferred neutrophils or an improved activation of other cell types involved in *S. pneumoniae* clearance, triggered by transferred neutrophils. Further studies aimed at understanding how neutrophils interact and shape the early response in the lungs against *S. pneumoniae* are needed.

The reduced lung histopathology observed in *Il10* ^-/-^ mice transferred with WT neutrophils following *S. pneumoniae* caught our attention and may be an important indicative of the role of IL-10-producing neutrophils in response to this microbe. Several studies have shown that during lung bacterial infection, a balanced inflammatory response that promotes an efficient pathogen clearance with a limited tissue injury is required (19, 70, 71). In this context, we have previously shown that the production of IL-10 is critical to reduce lung inflammation and to improve host survival, but an early production of this cytokine impairs bacterial clearance (22). This fact has also been observed in a recent study showing that natural killer (NK) cells play a crucial role in pneumococcal pneumonia through IL-10 production, promoting bacterial growth and invasion to other host tissues after infection (72). We have extended this knowledge, providing evidence that neutrophils are the main myeloid cell source of IL-10 in response to *S. pneumoniae* after 48 h of infection. Here we also show that IL-10 production after infection is important to reduce lung histopathology but has negative impact on the ability of the mouse immune system to control bacterial burden. We also show that an early neutrophil recruitment is critical to ensure host survival in a mechanism that is apparently independent of IL-10 production. This finding highlights the plasticity and the complex biology of neutrophils that still remain largely unexplored. Unbiased techniques based on flow cytometry or single-cell RNA-seq may be useful to study the role of different neutrophil subsets in human and mice during infection. In fact, a recent study identified 10 different subsets/cluster of neutrophils in peripheral blood mononuclear cells (PBMCs) in COVID-19 patients, with potential implications in disease severity (73). These differential clusters identified showed significant differences in gene expression, showing a co-existence between anti-inflammatory/suppressive neutrophils and pro-inflammatory neutrophils, providing a more complete perspective of these cells, and also highlights the complexity of neutrophils during infection. Given the data available, it is possible that, in our model, neutrophils play a dual role in the lungs that, on the one side, protect the tissue from excessive immunopathology through the production of IL-10 but on the other side are required for bacterial clearance and pneumonia resolution. Based on our previous study (22) and the data provided here, we believe that the prevention of exacerbated lung inflammation by IL-10 production might be established following the bacterial clearance to guarantee an effective host defense. In this line and considering that neutrophils produce IL-10 before bacterial elimination, we might explain why WT mice and *Il10* ^-/-^ mice transferred with WT neutrophils present reduced lung inflammation and increased lung bacterial burden and dissemination.

The role of IL-10 production by human neutrophils is controversial. Conversely, there is an active debate about the capacity of human neutrophils to produce IL-10 (74). The origin of this discussion relies on one study that show that human neutrophils are unable to produce IL-10 due to an inactive chromatin configuration that impairs the *Il10* transcription (75). However, recent studies incorporating analyses of circulating neutrophils, have successfully shown mRNA expression and increased levels of IL-10 in response to different pathogens and stimulus, and they also have described at least two different pathways that explain its production (30, 55, 76, 77). This evidence opens up the possibility that, to some extent, characterization data obtained in our work could be further studied in humans during pneumonia

In conclusion, our findings contribute to a better understanding of the modulatory role of neutrophils in response to lung bacterial infection. One of the main aims of this work was to provide new insights regarding the anti-inflammatory response of neutrophils during pneumococcal pneumonia during the first 48 h of the infection. Furthermore, here we provide new morphological and functional data of the different lung neutrophil subsets previously described in our laboratory. Additionally, our findings show that populations of neutrophils found in mouse lungs express IL-10 directly in response to *S. pneumoniae*, having a key role in the outcome of the pathological process. Finally, our *in vitro* and *in vivo* data determined the unrecognized role of IL-10-producing neutrophils in pneumococcal pneumonia. Several studies are needed to translate our findings to humans, although our data may constitute the first step that could eventually translate into novel therapeutic strategies that improve the management of patients with pneumococcal pneumonia and invasive pneumococcal disease.

## Data Availability Statement

The raw data supporting the conclusions of this article will be made available by the authors, without undue reservation.

## Ethics Statement

The animal study was reviewed and approved by Comité Ético Científico para el Cuidado de Animales y Ambiente, Pontificia Universidad Católica de Chile.

## Author Contributions

LG, FM-G, VS, OV, LN, BS, and IS performed the experiments. LG, FM-G, and SB designed the experiments. LG, FM-G, and SB analyzed the data. LG, FM-G, HP, CR, PG, DP, AK, and SB contributed to writing the paper. AM, DP, OV, and JS performed histopathological analyses. All authors contributed to the article and approved the submitted version.

## Funding

This study was supported by grants no. 1170964 (SB), 1190830 (AK), 1191300 (CR), and 1190864 (PG) from Fondo Nacional de Ciencia y Tecnología de Chile (FONDECYT), ANID-Millenium Science Initiative Program - ICN09_016: Millenium Institute on Immunology and Immunotherapy (ICN09_016; P09/016-F), grant no. 21150853 from the Agencia Nacional de Investigación y Desarrollo (LAG), grant no. 13CTI-21526 P5 from INNOVA-CORFO program of Chilean Ministry of Economy (SMB), the Innovation Fund for Competitiveness FIC- R 2017 (BIP Code: 30488811-0) a grant no. R01HL134870 from the NIH and Pilot Project Program in Hemostasis and Vascular Biology (P3HVB), University of Pittsburgh Vascular Medicine Institute, the Hemophilia Center of Western Pennsylvania, and the Institute for Transfusion Medicine (HFP).

## Conflict of Interest

The authors declare that the research was conducted in the absence of any commercial or financial relationships that could be construed as a potential conflict of interest.

## Footnotes

1 Yin, C & Fan C. (2012). Pneumococcal cell wall purification. http://www.bowdish.ca/lab/wp-content/uploads/2012/08/Pneumoncoccal-Cell-Wall-Purification-2DB-edits.pdf [Accessed August 5, 2020]
