# Supplementary material for: Characterization of the Anti-Inflammatory Capacity of IL-10-Producing Neutrophils in Response to Streptococcus pneumoniae Infection
Source: Front Immunol. 2021 Apr 28;12:638917. doi: 10.3389/fimmu.2021.638917 (PMC8113954; doi:10.3389/fimmu.2021.638917)
Supplement: Supplementary file 1 [file DataSheet_1.pdf]

Supplementary information

Parameter	Value	Weighted score	Maximum score
**Weight loss**	5%	0	4
	10%	1	
	15%	2	
	20%	3	
	25%	4	
**Activity**	Normal	0	4
	Slightly decreased	1	
	Decreased	2	
	Severely decreased	3	
	Coma	4	
**Time to return to upright position**	Normal	0	6
	< 5 seconds	2	
	< 30 seconds	4	
	No return is observed	6	
**Fur appeararance**	Normal	0	3
	Slightly dirty	1	
	Dirty	1	
	Piloerection	1	
**Posture**	Normal	0	2
	Slightly hunched over	1	
	Extremely hunched over	2	
**Eyes appearance**	Normal	0	4
	Protruding eyes	1	
	Sunken eyes	1	
	Closed eyelids	1	
	Ocular secretion	1	
**Breathing problems**	Irregular breathing	2	4
	Difficulty breathing	2	
**Neurological issues**	Normal	0	10
	Ataxia	2	
	Paralysis	2	
	Epileptic episodes	2	
	Status epilepticus	6	
**Total**			37

Table S1. Monitoring chart for infection caused by *Streptococcus pneumoniae.*

Score	Cell infiltrate	Hemorrhage	Swelling of alveolar walls	Lung damage	Damage extension
0	Normal amounts of alveolar macrophages; no presense of leukocytes in alveoles or bronchioles.	No presence of erithrocytes in alveoli	Absent	Normal lung architecture	No lessions are observed
1	Moderate increase of leukocytes in alveoli and/or bronchioles	Limited presence of erithrocytes in alveoli	Moderate	Minimal change in lung architecture	Focal unilateral lession
2	Severe increase of leukocytes in alveoli and/or brochioles	Elevated presence of erithrocytes in alveoli	Severe	Moderate change in lung architecture with alveoles and brochioles structures distinguishable	Focal and bilateral lessions/diffuse unilateral lession
3	Cellular infiltrate does not allow to recognize bronchioli structure	————	Indistinguisible structure	Any lung structure can be recognized	Diffuse lessions in both lungs

Table S2. Score of histopathological changes in lungs. The parameters included in the table were evaluated in representative photographs from complete lungs with the Aperio ImageScope Software. The score of microscopic lesions was determined at 20X magnification and the damage extension was evaluated at 4X.

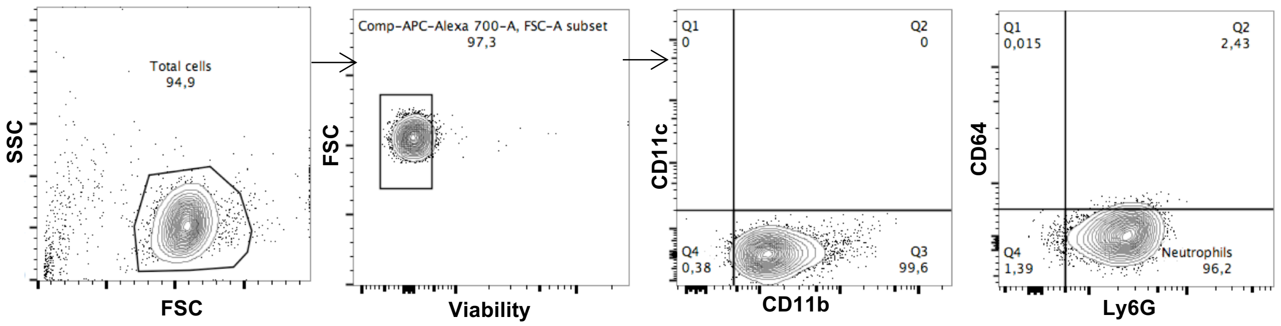

Figure S1. **Neutrophils purity after isolation from bone marrow cells.** Representantive gating strategy to determine neutrophils population and purity after the negative selection performed with MACS® columns.

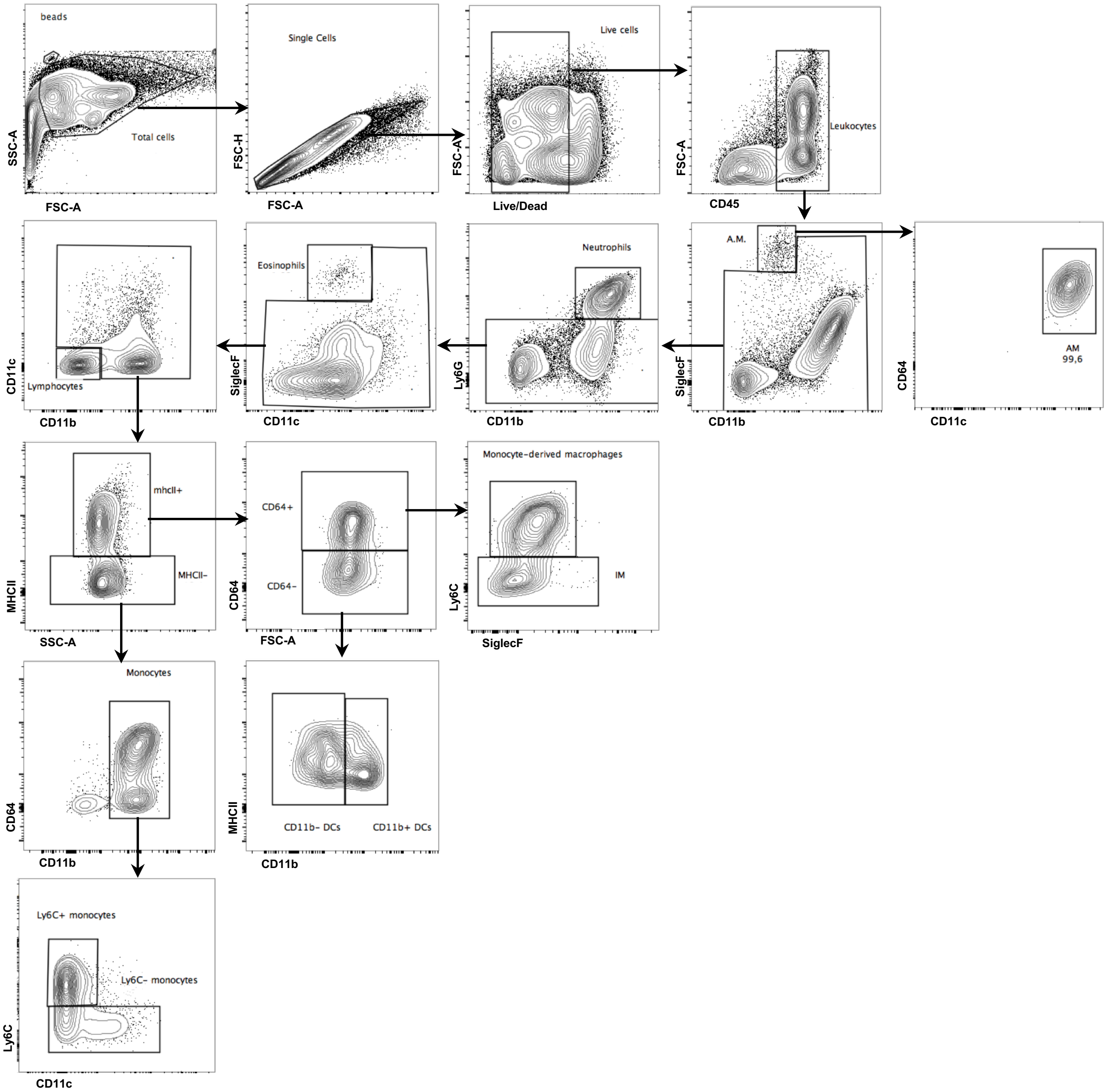

Figure S2. **Gating strategy for myeloid cell lines.** Viability was measured by staining with LIVE/DEAD fixable Viability Stain 510 (BD biosciences). The antibodies used to identify each cell population were: CD45-BV786 (BD), CD11b-PE (BD), Siglec-F-PE-CF594 (BD), Ly6G-Alexa Fluor 700 (Biolegend), CD11c-PE-Cy7 (BD), MHC-II-BV650 (BD), CD64-Alexa Fluor 647 (Biolegend), Ly6C-BV605 (BD). Counting beads (Life Technologies) were used to calculate the total number of cells in the lungs (usually diluted in 1ml). IM: Interstitial Macrophages; AM: Alveolar Macrophages

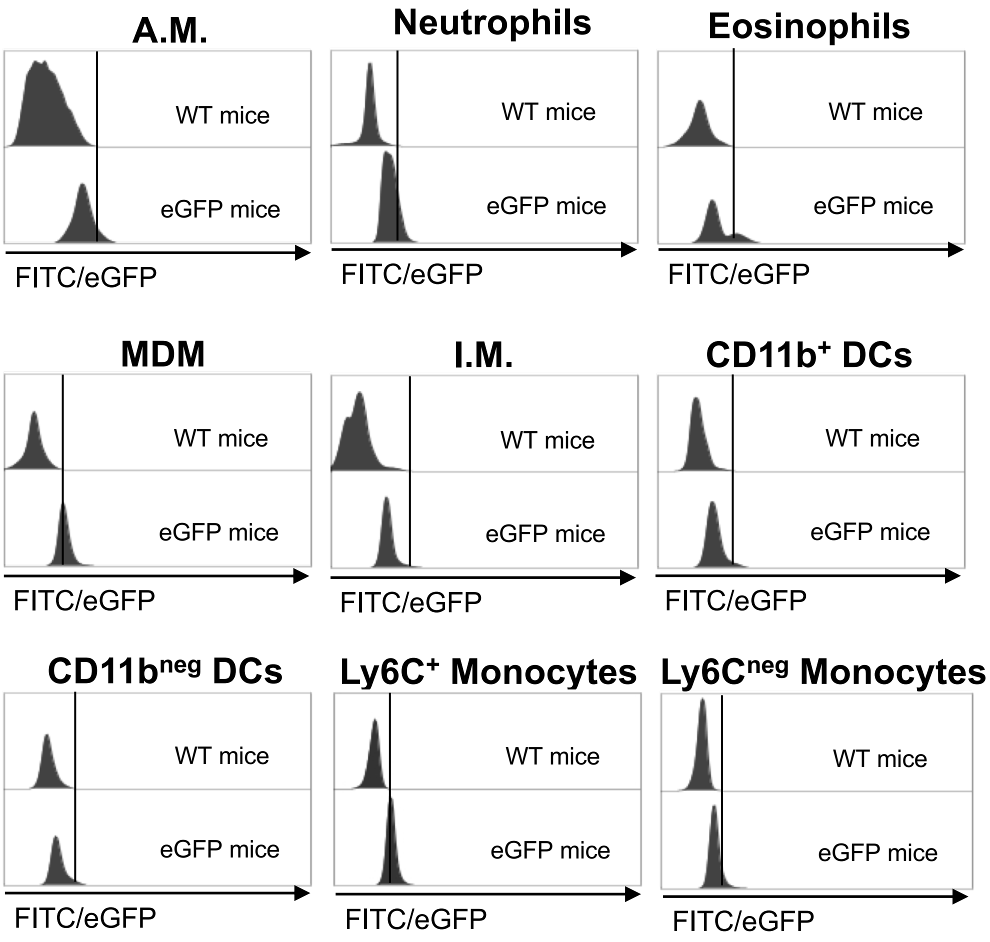

Figure S3. **Identification of autoflorescense in myeloid cells from the airways.** The basal eGFP expression by each cell population was determined using an uninfected WT (eGFP^{neg}) mice. IM: Interstitial Macrophages; AM: Alveolar Macrophages; MDM: Monocyte derived macrophages; DCs: Dendritic cells.

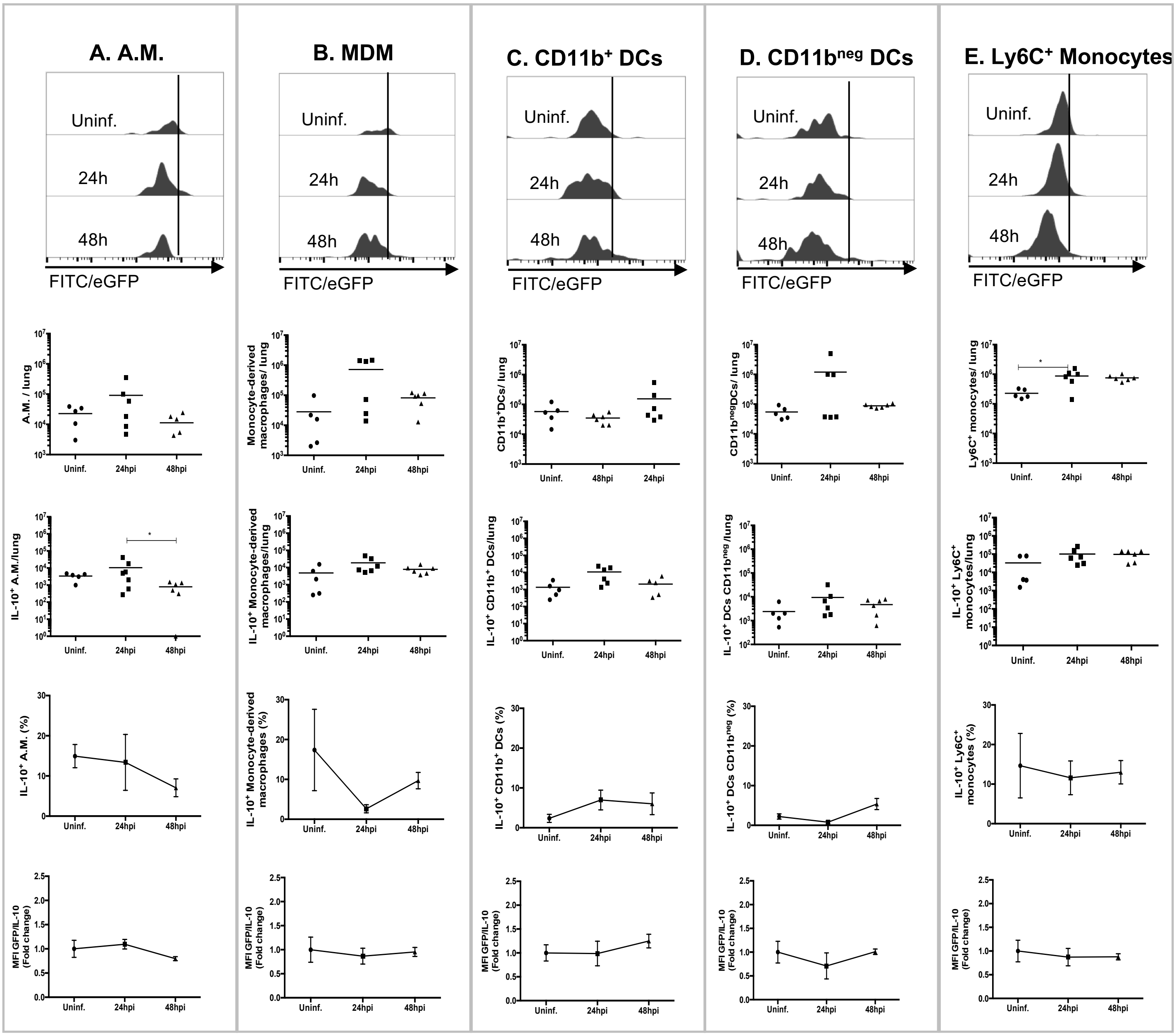

Figure S4. IL-10 production in alveolar macrophages, monocyte-derived macrophages and DCs. Number of cells expressing IL-10 in lungs in uninfected mice (n = 5), and mice infected after 24 (n = 6) and 48 hpi (n = 6) and their corresponding MFI measures. (A) Alveolar macrophages. (B) Monocyte-derived macrophages. (C) CD11b$^+$ DCs. (D) CD11b$^-$ DCs. (E) Ly6C$^+$ Monocytes. WT uninfected mice were used to establish the baseline of GFP expression (data not shown). Statistics: Kruskal-Wallis with Dunn's multiple comparison post-test. $P<0.05$. Comparisons of the mean of infected and uninfected mice, 24 and 48 hpi. hpi: h post-infection; MFI: Median Fluorescence Intensity; A.M.: Interstitial macrophages; MDM: Monocyte-derived macrophages; DCs: Dendritic cells.

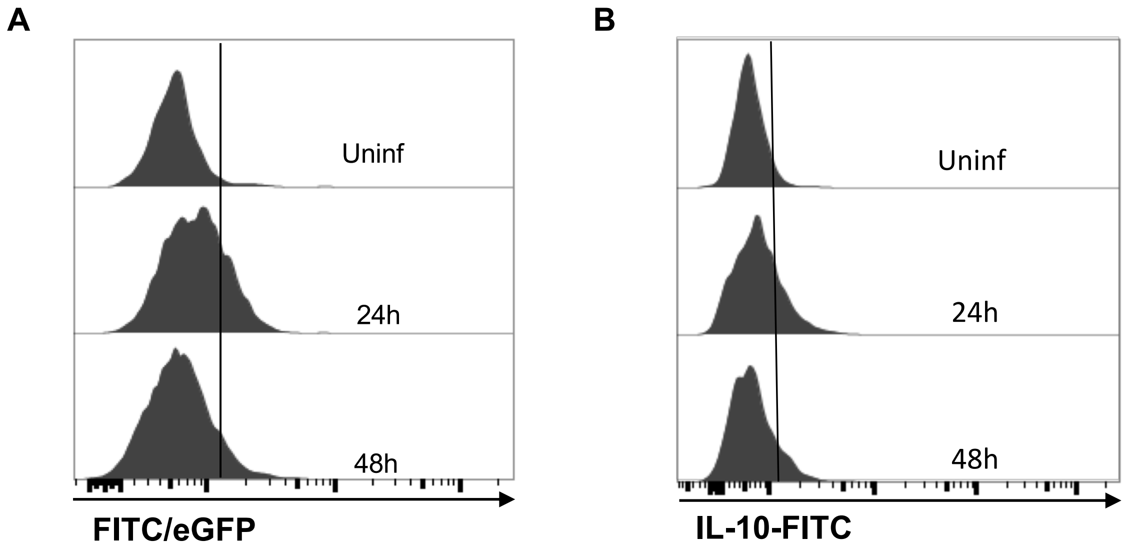

Figure S5. Comparison of IL-10 expression measured by eGFP fluorescence and IL-10 intracellular staining. Representative histograms of the IL-10 expression in neutrophils measured at 24 and 48hpi with *S. pneumoniae.* **A.** IL-10 expression measured in neutrophils from IL-10::eGFP mice. **B.** IL-10 expression determined with a FITC anti-IL-10 antibody. For the intracellular staining used in this protocol cells were stimulated using PMA (25ng/mL), ionomycin (375ng/mL), brefeldin A (0.5X) and monensin (0.5X) overnight at 37ºC in a CO_2 incubator. Cells were then stained for surface markers for 30 min, followed by fixation and permeabilization using the BD CytoFix/CytoPerm Fixation/Permeabilization kit (BD), according to the manufacturer's instructions. Cell were stained using a FITC-IL-10 antibody (clone JES5-16E3, Biolegend) for 1h at 4ºC.

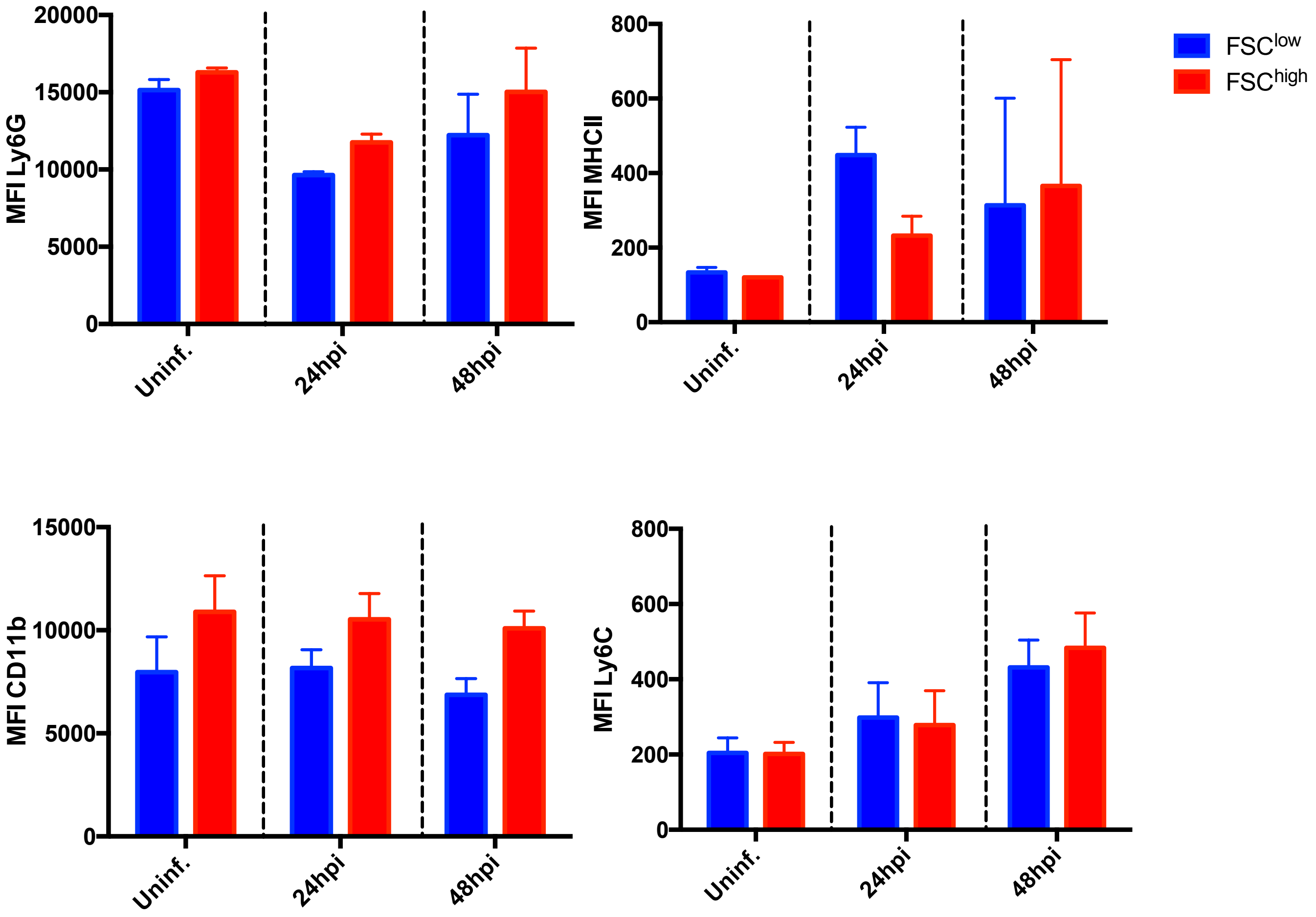

Figure S6. Activation and maturity surface markers are similar in FSC^{low} and FSC^{high} subtypes. Surface markers expression was evaluated by flow cytometry and was measured by median fluorescence intensity (MFI). Ly6G and Ly6C markers showed to be similar between the two neutrophil subtypes. CD11b and MHCII expression showed slight differences but no significant differences were evidenced. Statistics: Holm-Sidak test following two-way ANOVA. $p < 0.05$.

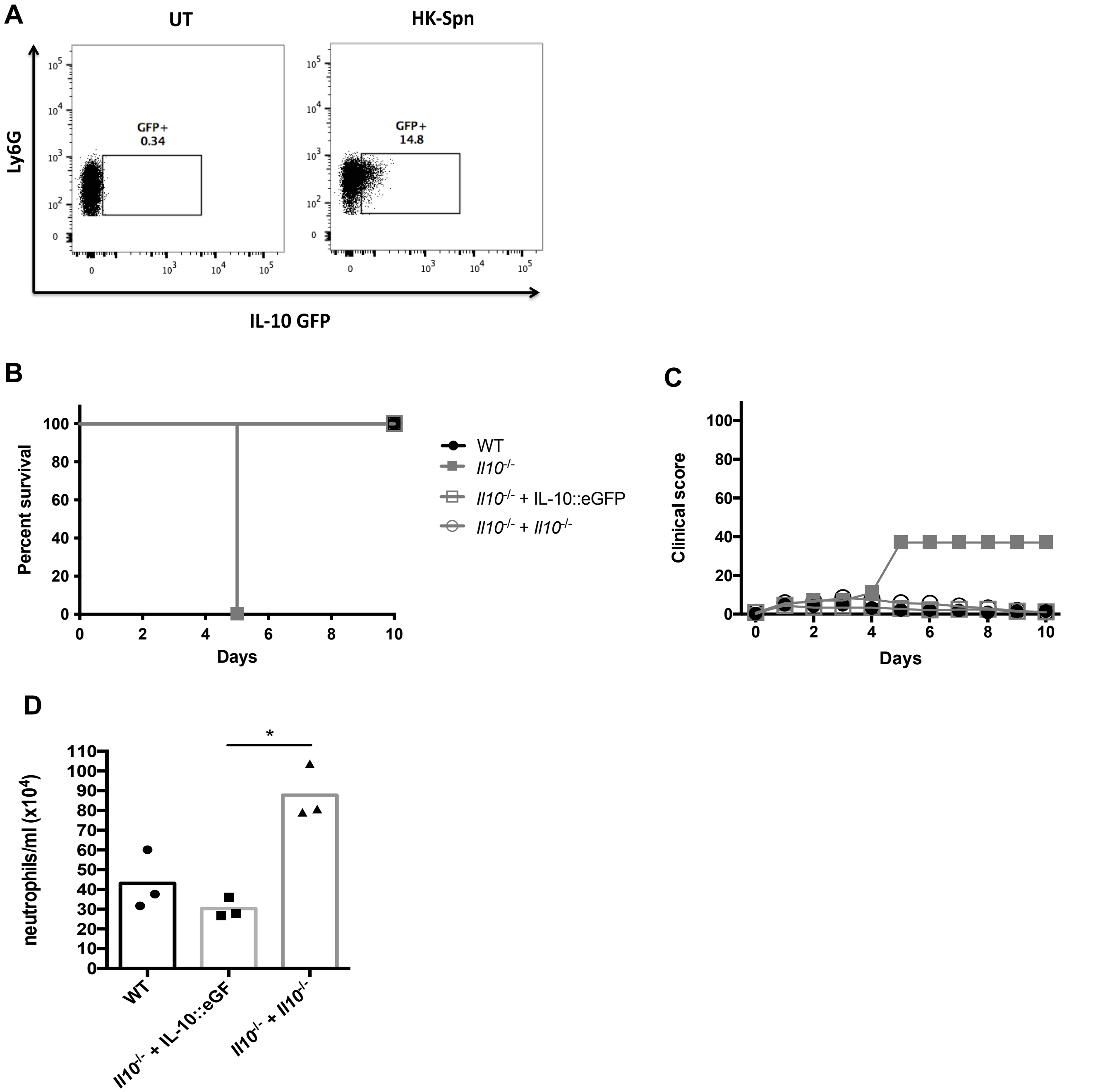

Figure S7. Neutrophils adoptive transfer improves *Il10*$^{-/-}$ mice survival independently of IL-10 production. Neutrophils obtained from IL-10::eGFP and *Il10*$^{-/-}$ mice were intranasally transferred to *Il10*$^{-/-}$ recipient mice 12 h after infection with $3x10^7$ CFU of heat-killed *S. pneumoniae*. Survival and clinical parameters of mice in the different groups were evaluated for 10 days. **A.** Neutrophils isolated from the bone marrow of IL-10::eGFP mice were stimulated for 12 h with heat killed *S. pneumoniae* (MOI=25) and the eGFP fluorescence was detected by flow cytometry in Ly6G$^+$ population. **B.** Survival curve of mice intranasally transferred with bone marrow derived neutrophils, previously stimulated with heat killed *S. pneumoniae*. **C.** Disease parameters were evaluated according to a clinical score guideline including activity, weight loss, body posture and general appearance. **D.** Neutrophils obtained from lungs of WT and transferred *Il10*$^{-/-}$ mice 10 days post-infection. Statistics: Log-rank (Mantel-Cox) test, Two-way ANOVA with Sidak's multiple comparisons post-test, and T test with Mann Whitney's post-test were performed. P < 0.05.

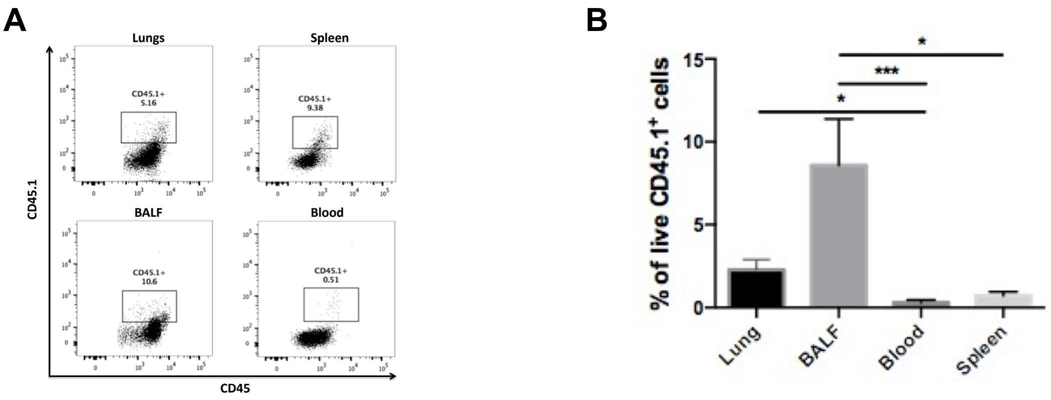

Figure S8. Distribution of intranasally transferred neutrophils in lungs, BALF, spleen and blood 48h post-transfer. Intranasally transferred CD45.1 neutrophils were tracked in lungs, BALF, blood and spleen 48 h post-transfer process in infected mice. **A.** Flow cytometry analysis of $CD45.1^+$ neutrophils in lungs, spleen, BALF and blood. Cells were gated as $CD45^+$ $CD11b^+$ $Ly6G^+$. **B.** Percentage of live CD45.1+ neutrophils from the total cell counts found in lungs, BALF, blood and spleen after 48h post-transfer. Statistics: One-way ANOVA with Dunn's multiple comparisons post-test were performed. $P < 0.05$.

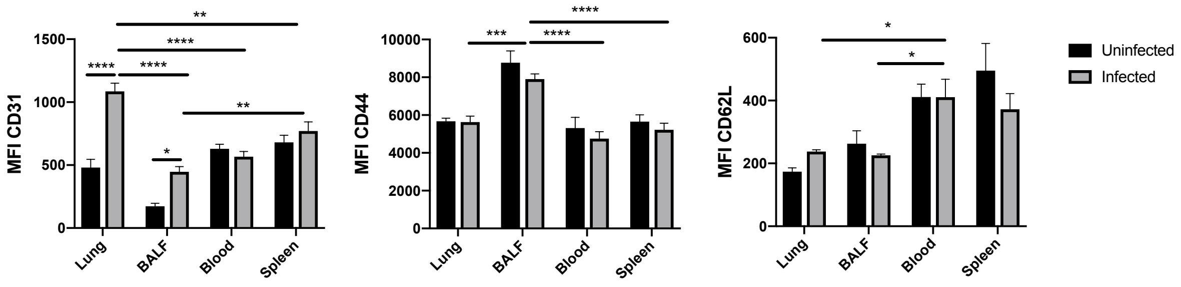

Figure S9. Surface homing markers in CD45.1 transferred neutrophils. The MFI of CD31, CD44 and CD62L was measured in CD45.1 neutrophils obtained from lungs, BALF, blood and spleen after 48 hours post-transfer. Statistics: Two-way ANOVA with Sidak's multiple comparisons post-test, and T test with Mann Whitney's post-test were performed. P < 0,05.

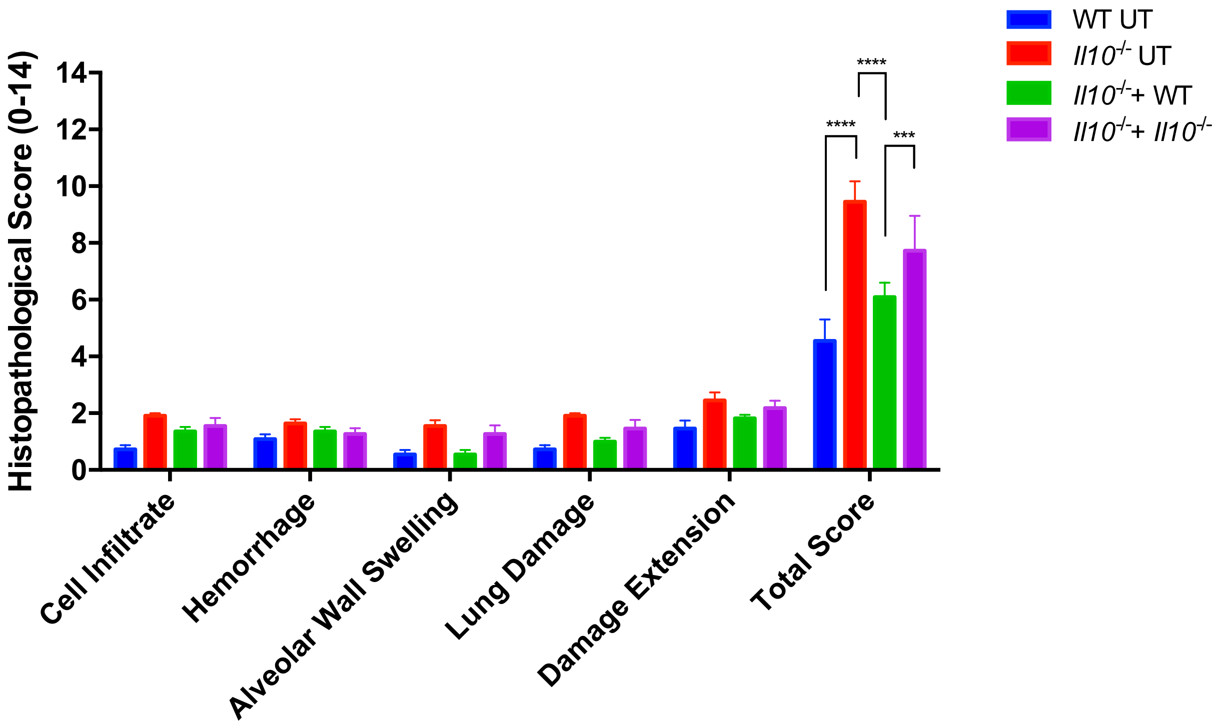

Figure S10. Complete histopathological score for lung damage. Five lung damage parameters were evaluated in representative photographs of complete lungs, to determine a complete score for severity of pneumonia. Statistics: Two-way Anova with Tukey's multiple comparisons post-test were performed. P<0.05.
